# Comprehensive Evaluation of the Elecsys HCV Duo Immunoassay for Active Hepatitis C Detection: Insights From the DEPCDUO Study

**DOI:** 10.1002/jmv.71050

**Published:** 2026-07-04

**Authors:** Paul‐Elie Gosset, Célia Margueron‐Sentis, Christophe Ramière, Fabien Zoulim, Caroline Scholtès

**Affiliations:** ^1^ Department of Virology Hospices Civils de Lyon Lyon France; ^2^ Université de Lyon, Lyon 1 Université Lyon France; ^3^ Lyon Hepatology Institute, IHU EVEREST Lyon France; ^4^ Department of Virology, INSERM Unit 1111, CIRI Hospices Civils de Lyon Lyon France; ^5^ Department of Hepatology, INSERM Unit 1350 PathLiv Hospices Civils de Lyon Lyon France; ^6^ Department of Virology, INSERM Unit 1350 PathLiv Hospices Civils de Lyon Lyon France

**Keywords:** early detection, Elecsys HCV duo, fourth‐generation assay, HCV core antigen, HCV elimination strategy, hepatitis C diagnosis

## Abstract

Early diagnosis of Hepatitis C virus (HCV) infection is a cornerstone of elimination strategies. Fourth‐generation antigen/antibody combination assays, such as Elecsys HCV Duo, promise to bridge the diagnostic gap left by antibody‐only testing. In this real‐world study, we assessed Elecsys HCV Duo performance using seroconversion panels and routine clinical samples, focusing on sensitivity, specificity, and positive predictive value (PPV) for active HCV detection, including challenging scenarios with low viral loads and HIV co‐infections. The assay shortened the serological window by up to 203 days. When both antigen and antibody were detected, PPV reached 100%, enabling confident diagnosis without immediate RNA confirmation. The antigen component alone achieved a PPV of 97.6%, even in a low‐prevalence setting (0.33%), better than previously reported assays. However, sensitivity for low viral loads (≤ 5 log_10_ IU/mL) was limited, with 95.5% of such cases missed. Elecsys HCV Duo offers a powerful tool for earlier detection and streamlined care, particularly in high‐risk and resource‐limited contexts. Yet, confirmatory RNA testing remains essential for low viral load cases. Broader validation is needed to confirm its utility in diverse populations, reinfection surveillance, and cost‐effective screening strategies.

## 1 Introduction

Hepatitis C virus (HCV) infection remains a significant global health concern. In 2022, the World Health Organization (WHO) estimated that approximately 50 million people were living with HCV, with an annual incidence of 1 million cases [1]. Among those infected, about 70% develop chronic infection, which progresses to cirrhosis in 10–20% of cases and increases the annual risk of hepatocellular carcinoma (HCC) by 1%–4% if left untreated. These complications contribute to over 242,000 deaths globally each year [2].

HCV diagnosis in France follows a two‐step process: initial detection of anti‐HCV antibodies (HCVAb) to determine exposure, followed by nucleic acid amplification tests (NAATs) to confirm active infection [3]. Antibody detection is commonly performed using third‐generation serological tests, which identify total HCVAb (IgM and IgG) [4]. However, these tests face several limitations. The antibody detection window is long, typically 6–12 weeks post‐infection, and may extend beyond 12 months in immunocompromised individuals, including people living with HIV (PLWHIV) [5, 6]. Moreover, serological tests cannot distinguish between active and resolved infections. NAATs, while reliable for detecting active infections, are costly, time‐intensive, and require specialized expertise and equipment.

Early and accessible HCV detection is critical for curbing the epidemic, particularly among high‐risk populations such as PLWHIV and people who inject drugs. In these groups, delayed seroconversion and barriers to healthcare access pose diagnostic challenges [7]. The WHO has prioritized eliminating hepatitis C as a public health threat by 2030, emphasizing improved screening strategies to identify individuals with chronic HCV infection. This is particularly pressing as only 36% of HCV‐infected individuals worldwide are estimated to be aware of their serological status [1]. Given that HCV is largely asymptomatic until advanced stages, there is an urgent need for simple, cost‐effective, and reproducible methods to identify active infections.

HCV core antigen (HCVcAg) detection has emerged as a promising alternative to NAATs [8]. HCVcAg can be detected in peripheral blood within 12–15 days of infection, approximately 1–2 days after HCV RNA, significantly shortening the diagnostic window compared to antibody testing alone [9]. Recognized as a reliable marker of viral replication, HCVcAg detection is endorsed by the European Association for the Study of the Liver (EASL) as a viable substitute for NAATs (grade A1) [8]. Recent studies, including a meta‐analysis by Sepulveda‐Crespo et al., have demonstrated the strong analytical performance of the Abbott Architect HCV Antigen test for diagnosing active HCV infections [10]. However, simultaneous testing with the HCVAb assay on the same Architect can result in false positives, highlighting the need for careful evaluation of combined diagnostic approaches.

Fourth‐generation serological tests that detect both HCVcAg and HCVAb in parallel offer an opportunity to address these challenges. Such tests not only reduce the diagnostic window but also minimize reliance on molecular testing, which may be inaccessible or prohibitively expensive in resource‐limited settings. The Elecsys HCV Duo test (Roche Diagnostics GmbH, Mannheim, Germany) is an electrochemiluminescence immunoassay (ECLIA) designed to simultaneously detect HCVcAg and HCVAb in separate, parallel reactions within 27 min. Compatible with serum, heparinized plasma, or EDTA, this CE‐marked test provides results expressed as an index, with the ability to identify which marker is reactive in positive cases. A previous study by Majchrzak et al. showed that this test had a specificity of 99.9% and a sensitivity of 99.6%, similar or better than competitors' third or fourth‐generation assays for HCV screening.

In the present study, the primary aim was to evaluate the performance of the Elecsys HCV Duo test under real‐world conditions, with a particular focus on the “antigen” module's ability to identify active HCV infections. Additionally, the assay was retrospectively assessed on plasma samples with low viral loads to further investigate its diagnostic sensitivity.

## 2 Materials and Methods

### 2.1 Study Design

This study evaluated the results obtained with the Elecsys HCV Duo test during routine implementation at the Hospices Civils de Lyon (HCL) virology laboratory over a 6‐month period (May 2, 2023, to November 2, 2023). The Elecsys HCV Duo assay (Roche Diagnostics) is an in vitro diagnostic, sandwich‐type electrochemiluminescence immunoassay (ECLIA) performed on the cobas e801 analyzer (Roche Diagnostics), intended for the simultaneous qualitative detection of hepatitis C virus (HCV) capsid antigen and total antibodies to HCV in human serum or plasma. HCV capsid antigen is detected using biotinylated and ruthenylated monoclonal antibodies directed against the HCV core protein, while total anti‐HCV antibodies are detected in a separate reaction channel using recombinant HCV antigens (core, NS3, and NS4 proteins). Results are expressed as a cut‐off index (COI), with values ≥ 1.0 considered reactive and values < 1.0 considered non‐reactive, according to the manufacturer's instructions. A total of 24,656 tests were conducted, of which 22,731 unique results were included after excluding multiple concordant screenings from the same patient. The study protocol was designed and reported in accordance with the STARD (Standards for Reporting Diagnostic Accuracy Studies) guidelines, approved by the local ethics committee (approval no. 24‐5089), and conducted in accordance with the Declaration of Helsinki and applicable national regulations.

All antigen‐positive samples were subjected to quantitative reverse transcriptase polymerase chain reaction (RT‐PCR) for HCV RNA detection (cobas HCV, Roche Diagnostics GmbH, Mannheim, Germany), which served as the reference method for confirming viral replication. Samples with newly detected HCVAb alone were also tested for HCV RNA. When feasible, HCV genotyping was performed using the ViroKey SQ FLEX system (VELA Diagnostics GmbH, Hamburg, Germany). Additional clinical and biological data, including patient age, hepatitis type (acute or chronic), alanine aminotransferase (ALT) levels, symptomatology, risk factors, HIV co‐infection status, treatment with direct‐acting antivirals (DAAs), HCV genotype, hepatic fibrosis or cirrhosis, hepatocellular carcinoma (HCC), hepatic transplantation (HT), and the prescribing service, were collected for positive cases.

During the study period, a total of 1340 viral loads (VLs) were performed, including 749 samples with associated serology. For viral loads performed without accompanying serology, 41 positive VL samples were identified and subsequently tested using the Elecsys HCV Duo assay. Due to insufficient sample volume, three samples could not be tested.

Additionally, the Elecsys HCV Duo test's reactivity for its two components (HCVcAg and HCVAb) was evaluated using samples collected between April 2022 and May 2023 with a viral load (VL) ≤ 5 log_10_ IU/mL (*n* = 21).

To compare screening performance with the third‐generation HCVAb test previously used in the laboratory (Atellica aHCV, Siemens Healthcare Diagnostics, Munich, Germany), sera from the laboratory's “seroconversion panel” (*n* = 19) were tested using the Elecsys HCV Duo assay. The “seroconversion panel” refers to patients who initially tested negative using a third‐generation anti‐HCV assay and were subsequently diagnosed with active HCV infection during follow‐up. The analyzed samples correspond to the last specimen, testing negative for total anti‐HCV antibodies with the third‐generation assay, and the first specimen in which active infection was detected. Sera that were negative with the 3rd‐generation test and positive with the 4th‐generation test were subsequently tested by RT‐PCR to confirm the presence of active infection missed at the first timepoint.

The ability of the Elecsys HCV Duo test to detect reinfections was also assessed in cases identified during the study period, defined as reappearance of detectable HCV RNA after a previously documented sustained virological response (SVR) in patients with ongoing risk of HCV exposure.

### 2.2 Statistical Analysis

Statistical analyses were performed using GraphPad Prism 10 software. Descriptive statistics were expressed as counts and percentages. Differences in means for antigenic indexes or age across subpopulations were assessed using Student's *t*‐test. A *p* value < 0.05 was considered statistically significant.

The sensitivity and specificity of the Elecsys HCV Duo test for detecting active HCV infection were evaluated against viral load results, with true positives defined as samples with HCVcAg levels > 1 and confirmed by HCV RNA. A Receiver Operating Characteristic (ROC) curve analysis was performed to determine the optimal antigenic index threshold for interpreting results in patients with anti‐HCV antibodies.

## 3 Results

**Figure 1:**
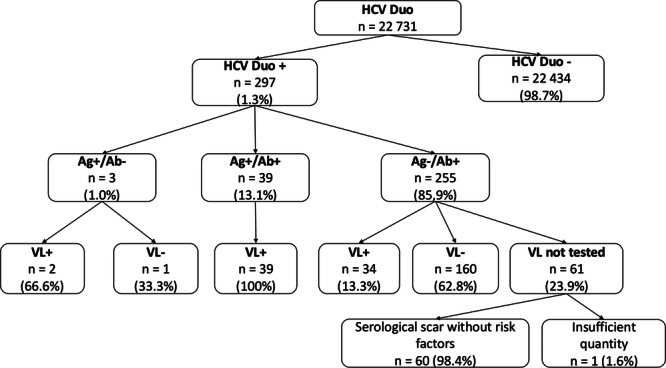
Overview of Elecsys HCV Duo screening and confirmatory test results. + positive, − negative; Ag Core Antigen; Ab HCV antibodies, VL viral load.

Over a 6‐month period, 24,656 Elecsys HCV Duo tests were conducted at the Hospices Civils de Lyon, a tertiary hospital encompassing all clinical specialties. After excluding 1925 duplicate tests, 22,731 unique analyses were included. Approximately 30% of test prescriptions originated from screening centers such as CeGIDD (free screening center for sexually transmitted illnesses) or infectious disease departments, with smaller contributions from hepato‐gastroenterology (4.2%), addictology/psychiatry (3.9%), and hematology/oncology departments (9%). The median age of tested individuals was 37 years (mean: 42.3 years, range: 0–101 years). Among the unique tests, 297 (1.3%) were positive, with the majority (85.9%, *n *= 255) showing antibody positivity alone. Antigen‐only positivity was rare (1.0%, *n *= 3), while 13.1% (*n *= 39) were positive for both antigen (Ag) and antibody (Ab) (Figure 1).

Biological characteristics of the tested samples are summarized in Table 1. Patients with active HCV infection were significantly older (mean: 48.3 years, median: 49.5 years) than those with negative screening results (mean: 42.2 years, median: 37 years, *p* = 0.0088).

**Table 1:** Biological characteristics of samples tested with the Elecsys HCV duo test.

	Ag+/Ab−	Ag+/Ab+	Ag−/Ab+	Ag−/Ab−
	(*n* = 3)	(*n* = 39)	(*n* = 255)	(*n* = 22 434)
Mean antigenic index +/− standard deviation	88.5 +/− 146.8	9.9 +/− 13.7	0.56* +/− 0.07	0.54 +/− 0.09
Median antigenic index	4.8	4.1	0.54	0.53
(IQR)	(2.8–258)	(1.9–12)	(0.52–0.57)	(0.52–0.56)
Mean HCV VL l log_10_ UI/mL +/− standard deviation	7.0 (*n* = 2) +/− 1.7	6.3 (*n* = 39)	5.04 (*n* = 34) +/− 1.1	NA
		+/− 0.7		
Median HCV VL log_10_ UI/mL	7.0	6.3	5.2	NA
(IQR)	(5.8–8.2)	(5.8–6.8)	(4.5–5.7)	
Age (mean)	40.7	48.9	54.7	42.1
HIV co‐infection (%)	2 (66.6%)	3 (7.7%)	19 (7.4%)	1102 (4.9%)
Genotype				
1a	1	17	3	NA
1b	0	7	1	
2	0	3	1	
3a	0	5	1	
4	1	3	1	
Not performed	0	4	24	

Abbreviations: Ab = antibody, Ag = antigen, NA = not available.

*in the case of active infection, mean antigenic index: 0.65 (*p* value < 0.0001) (*n* = 34).

### 3.1 Antigen and Antibody Results

In this study, 9 samples were initially found positive for HCV Ag alone. However, after reanalysis following the manufacturer's recommendations, only three samples retained an index value above the positivity threshold of 1. Among the six samples initially reactive for HCV antigen but non‐reactive upon repeat testing, antigen index values were close to the positivity threshold. The median antigen index decreased from 1.3 (IQR: 1.1–4.0) at first testing to 0.5 (IQR: 0.5–0.5) upon repeat analysis, consistent with borderline reactivity and suggesting initial false‐positive results. No specific common characteristic was identified. HCVcAg values of those remaining positive after retest ranged from 2.82 to 258. Two of these 3 showed a positive viral load (VL). Notably, none of these patients exhibited hepatic cytolysis.

All 39 samples with dual Ag+/Ab+ results corresponded to active infections (confirmed HCV RNA‐positive), with mean and median viral loads of 6.3 +/− 0.7 log_10_ IU/mL (IQR: 5.8–6.8 log_10_ IU/mL). Interestingly, only 53.8% (*n* = 21) of these patients exhibited elevated ALT levels (mean ALT: 9.1 times the upper normal limit).

Among antibody‐only positive samples (*n* = 255), 13.3% (*n* = 34) had active infections, as evidenced by positive HCV RNA. Mean HCV RNA level of these samples with negative HCV Ag was 5.04 +/− 1.1 log_10_ IU/mL (median 5.19 log_10_ IU/mL, IQR: 4.5–5.7).

Although negative according to the manufacturer's threshold, these samples exhibited a higher mean antigenic index (0.65) compared to cured or resolved cases (0.54, *p* < 0.0001).

**Figure 2:**
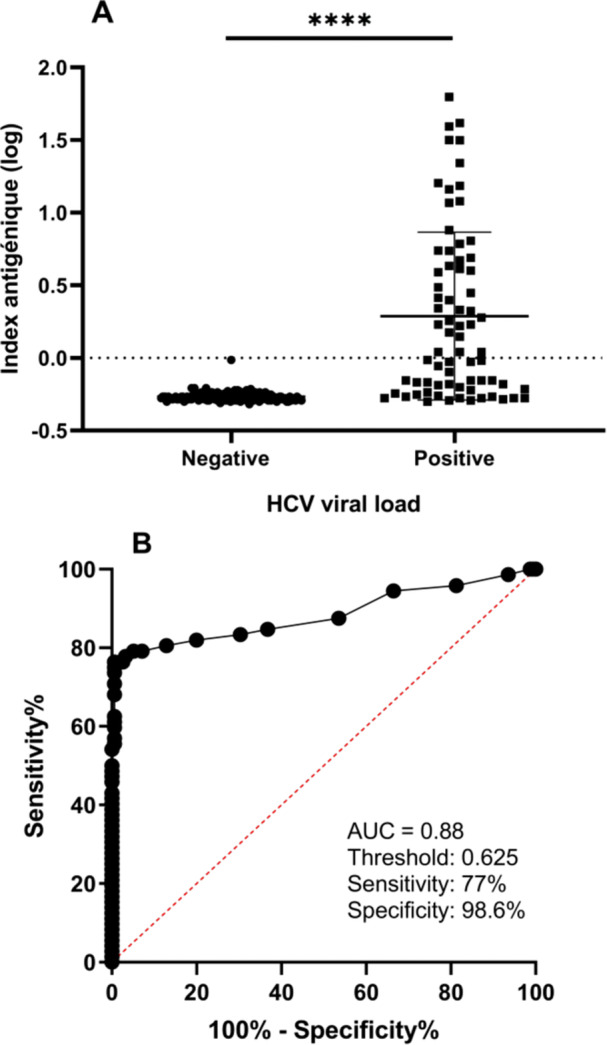
Antigenic index analysis in antibody‐positive patients. (A) Comparison of antigenic index values (log_10_) in antibody‐positive patients according to viral load results. Dotted line corresponds to the Elecsys threshold of positivity. (B) ROC curve: determination of an antigenic index threshold in antibody‐positive patients to identify active infection. Statistical significance of the antigenic index between samples with negative and positive viral loads was determined using Student's *t*‐test (****P < 0.0001).

Overall, for patients with antibody‐positive results (*n* = 294), there was a significant difference between the antigenic index of HCV‐positive and HCV‐negative viral load (*p *< 0.0001, Figure 2A). Moreover, in order to determine an antigenic index threshold for detecting active infections in antibody‐positive individuals, a ROC analysis identified a threshold of 0.625 with a sensitivity of 77% and a specificity of 98.6% (Figure 2B).

In total, 1126 of the patients who were subjected to serological testing (*n* = 22,731) were identified as being infected with HIV. Of these, 24 exhibited positive‐HCV serology (2.1%).

All samples with a positive viral load (*n* = 41) without serology initially requested were also screened as positive by Elecsys HCV Duo. Among these, 48.7% had a positive antigenic valence (*n* = 20), with a mean antigenic index of 14.9 +/− 20.9 (median: 5.2, IQR: 1.7–16.2) and negative (*n* = 18) with a mean antigenic index of 0.7 +/− 0.1 (median: 0.65, IQR: 0.57–0.83). The overall mean VL for these samples was 5.4 +/− 1.5 log_10_ IU/mL (median: 5.9 log_10_ IU/mL, IQR: 4.7–6.4).

Overall, the Elecsys HCV Duo assay demonstrated 99.9% specificity and 100% sensitivity for detecting HCV infection. However, focusing on the ability of the antigenic‐valence to detect active HCV infection, sensitivity dropped to 54.6% but increased to 70.9% for viral loads above 5 log_10_ IU/mL.

### 3.2 Low Viral Load

Between April 2022 and May 2023, 12% of HCV‐positive VLs were below 5 log_10_ IU/mL (mean 3.0 +/− 1.0 log_10_ IU/mL, *n* = 21). Low VL was associated with factors such as ongoing DAA therapy, acute hepatitis profiles, or underlying conditions like cirrhosis or dialysis. All samples were Ab‐positive but only one HCV Ag‐positive. Consequently, the sensitivity of the Elecsys HCV Duo assay for detecting HCV infection in cases with low viral loads (≤ 5 log_10_ IU/mL) was 100%, but only 4.5% to identify active HCV infection.

Among the 20 patients with low viral load and negative HCV‐Ag test results, clinical characteristics were heterogeneous. Immunocompromised conditions were present in 8 patients (40%), including solid organ transplantation, chronic hemodialysis, and malignancies. HIV infection was identified in 3 patients (15%). A history of intravenous drug use or substance use disorder was reported in 5 patients (25%). Acute hepatitis or acute HCV infection was observed in 3 patients (15%), while 2 cases (10%) were incidentally diagnosed during unrelated clinical evaluations. One patient (5%) had a long‐standing untreated chronic HCV infection. Several of these clinical contexts are associated with low‐level viremia or altered antigen kinetics, which may account for the negative antigen detection despite detectable HCV RNA.

**Figure 3:**
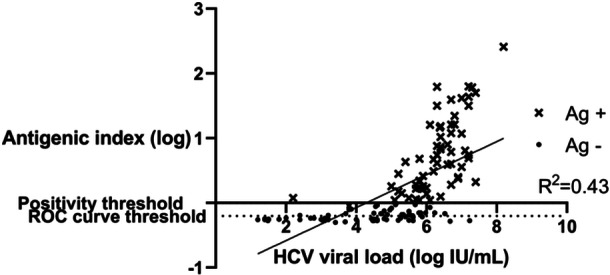
Correlation between HCV viral load (log_10_ UI/mL) and antigenic index (log_10_) of Elecsys HCV Duo assay.

Additionally, the study demonstrated a moderate correlation between viral load and the antigenic index (*r* ^2^ = 0.43). Notably, capsid antigen was more frequently (68%) positive when viral loads exceeded 5 log_10_ IU/mL (Figure 3).

### 3.3 Characteristics of HCV Infections

A total of 66 cases of chronic HCV infection were identified, with half of them displaying Ag−/Ab+ profiles. Most chronic cases were asymptomatic and often linked to intravenous drug use (Table 2a).

**Table 2:** Clinico‐biological characteristics of patients with chronic (a) and acute (b) active HCV infections.

a	Chronic hepatitis C	
	Ag+ /Ab+ (*n* = 32)	Ag−/Ab+ (*n* = 34)
Mean antigenic index +/− standard deviation	8.5 +/− 10.8	0.7 +/− 0.1
Median antigenic index (IQR)	4.1 (1.9–10.9)	0.6 (0.53–0.7)
Mean HCV VL log_10_ IU/mL +/− standard deviation	6.3 +/− 0.6	5.0+ /− 1.1
Median HCV VL log_10_ IU/mL (IQR)	6.3 (5.9–6.7)	5.2 (4.5–5.7)
Sex‐ratio (M/F)	2.5	2.7
Age mean (years)	52.4	47.8
ALT		
*N*	15 (46.8%)	20 (58.8%)
> *N*	14 (43.8%)	14 (41.2%)
Not available	3 (9.4%)	0
Clinical features		
No symptoms	28 (87.5%)	24 (70.6%)
Jaundice, abdominal pain	2 (6.25%)	1 (2.9%)
Cirrhosis, fibrosis	0	5 (14.7%)
Others (including HCC)	2 (6.25%)	1 (2.9%)
Not available	0	3 (8.8%)
Risk factors		
IDU	19 (59.4%)	14 (41.2%)
Unprotected sex	5 (15.6%)	2 (5.8%)
HIV	3 (9.4%)	1 (2.9%)
Genotype		
1a	12 (37.5%)	5 (14.7%)
1b	7 (21.9%)	1 (2.9%)
2	3 (9.4%)	2 (5.9%)
3a	5 (15.6%)	4 (11.8%)
4d	1 (3.1%)	2 (5.9%)
Co‐infection 1a,3a	0	1 (2.9%)
Not realized	4 (12.5%)	19 (55.9%)

b	Acute HCV infection		
	Ag+/Ab− (*n* = 2)	Ag+/Ab+ (*n* = 7)	Ag−/Ab+ (*n* = 4)
Mean antigenic index +/− standard deviation	131.4 +/− 179	16.6 +/− 23.0	0.7 +/− 0.2
Median antigenic index (IQR)	131.4 (4.8–258)	4.3 (1.5–31.6)	0.7 (0.55–0.9)
Mean HCV VL log_10_ IU/mL +/− standard deviation	7.0 +/− 1.7	6.1 +/− 1.0	4.4 +/− 0.8
Median HCV VL log IU/mL (IQR)	7.0 (5.8–8.2)	5.4 (5.2–7.2)	3.9 (3.5–4.9)
Age mean (years)	48.5	35.3	34.5
ALT			
*N*	2 (100%)	0	1 (25%)
> *N*	0	7 (100%)	3 (75%)
Clinical features			
No symptoms	2 (100%)	3 (37.5%)	1 (25%)
Jaundice, abdominal pain	0	2 (28.6%)	2 (50%)
Cirrhosis, fibrosis	0	0	0
Others	0	2 (25%)	0
Genotype			
1a	1 (50%)	5 (71.4%)	4 (100%)
4d	1 (50%)	2 (25%)	0

Overall, 14 cases of acute HCV infection (defined as a positive HCV serology and/or positive HCV VL in patients with a negative history less than 6 months old) were identified during the study period (Table 2b). All of these patients were male (mean age: 39 years) and exhibited high‐risk factors for HCV infection such as high‐risk behaviors (men who had unprotected sex with men or chemsex. Forty three percent were coinfected with HIV. Seven cases were identified with dual Ag+/Ab+ profiles, while two were Ag+ only. In five cases, acute infection was detected via HCV RNA direct testing due to suggestive clinical symptoms following high‐risk behavior. Subsequent serology on these plasmas was Ab‐positive in all 5 cases, but only one for the Ag valence with an index value ranging from 0.53 to 0.93. These patients had significantly lower HCV VLs than antigen‐positive patients (4.6 +/− 0.91 log_10_ IU/mL vs. 6.3 +/− 1.1 log_10_ IU/mL, *p* = 0.002, but the majority was infected with genotype 1a as for HCV Ag‐positive patients.

Importantly, genotypic analysis revealed that the Elecsys HCV Duo test effectively detected genotypes 1 through 4, without differences in serological profiles between antigen‐positive and “false” antigen‐negative results. This suggests that the antigenic detection component of the Elecsys HCV Duo test is capable of reliably identifying various HCV genotypes circulating in France.

### 3.4 Seroconversion Panel

Retrospective analysis of previously identified acute infections showed that the Elecsys HCV Duo would have been negative on its Ab‐valence in all samples, such as the third‐generation test used at the time of screening, but positive for the Ag valence in 12 of the 19 samples, with an antigenic index ranging from 1.7 to 468. Among these 12 samples, 9 were retrospectively confirmed with a positive VL for HCV, confirming the true positivity of the Elecsys HCV test. Six out of the 12 patients had normal ALT. Use of the Elecsys HCV Duo test would have enabled earlier detection of infection in 63.2% of cases, with a median time difference of 78 days (IQR: 43–140 days), based on the timing of available serial samples.

Three cases of HCV reinfection were identified in patients with a previously documented sustained virological response, occurring after a delay of 2.5–7 years (Table 3). At the time of reinfection, HCV RNA levels ranged from 2.1 to 6.4 log_10_ IU/mL. HCV core antigen was detected in 2 of the 3 cases, both associated with high viral loads (6.3 and 6.4 log_10_ IU/mL), whereas the third case, with low‐level viremia (2.1 log_10_ IU/mL), was antigen‐negative. All patients had ongoing risk factors for HCV exposure, including HIV co‐infection with high‐risk sexual behavior or intravenous drug use.

**Table 3:** Clinical and virological characteristics of HCV reinfection cases.

Patient	Time since cure	HCV RNA (log_10_ IU/mL)	Core Ag index	Anti‐HCV Ab index	First genotype/second genotype	Ongoing exposure
R1	7 years	2.1	0.53	17.2	4d/1a	HIV co‐infection; high‐risk MSM
R2	2.5 years	6.4	10.4	114	4d/4d	HIV co‐infection; high‐risk MSM
R3	4 years	6.3	5.47	18	Data not available/1a	Intravenous cocaine use

## 4 Discussion

Early diagnosis of HCV infection is essential to limit transmission and improve patient outcomes. Traditional HCV antibody‐based assays suffer from a prolonged serological window, limiting their ability to detect infections at an early stage. Fourth‐generation combined antigen/antibody assays, such as Elecsys HCV Duo, address this gap by simultaneously detecting HCVcAg and HCVAb.

In our real‐life evaluation, the Elecsys HCV Duo assay identified infection earlier than conventional serology in a subset of patients, with a maximal observed time difference of 253 days. However, this estimated gain must be interpreted with caution, as it depends on the timing and frequency of serial sample collection and reflects a theoretical difference based on retrospective data. Nevertheless, these findings are consistent with previous reports and support the potential utility of the assay for earlier diagnosis and improved linkage to care, particularly in high‐risk populations such as people who inject drugs or immunocompromised patients, including those living with HIV.

Dual Ag/Ab positivity demonstrated excellent reliability for active infection (PPV = 100%), supporting direct initiation of DAA therapy without systematic confirmatory RNA testing, as also observed by Bui et al. [11]. In contrast, Ag‐only results required confirmation due to occasional false positives, consistent with manufacturer recommendations. Our false‐positive rate (*n* = 1/22,731) was lower than that reported by Majchrzak et al. (13/20,634), underlining the assay's high specificity [12]. Considering only the positive results, the false‐positive rate was 1/297 (0.33%). The lower false‐positive rate observed in the present study may be attributable to better control of the pre‐analytical phase in a level 3 hospital compared with the multicenter study. Similarly, a meta‐analysis by Sepulveda‐Crespo et al. showed that HCV Ag assays may have a low PPV (< 59%) in settings where the prevalence of active infection is below 1%, necessitating confirmatory HCV RNA testing to ensure diagnostic accuracy [10]. On the other hand, it exceeded 80% in high‐prevalence populations (≥ 5%). The prevalence of active hepatitis C in the study was about 0.33%. According to the meta‐analysis, with this prevalence of active hepatitis C, the probability of the test being a true positive should be between 12% and 42%. A PPV of 97.6% was observed for the detection of antigen valence, which is not consistent with their results but might be explained by our lower sensitivity. EASL [8] and WHO guidelines support HCV‐Ag as a screening alternative to RNA in resource‐limited settings, but emphasize confirmation where prevalence is low or clinical suspicion persists. However, this recommendation is based on the high negative predictive value of the HCV Ag test alone [10]. The use of combined techniques to replace NAATs should be approached with caution. Moreover, a recent study investigated the role of HCVcAg results in assessing SVR instead of HCV viral load. They obtained a good specificity (97.4%) and a low false positivity rate (1.57%) but a relatively low sensitivity (57.1%), which might be explained by the difference of sensitivities in detecting viremia. Thus, HCV‐Ag testing could be helpful for assessing SVR after DAA therapy, with a HCV viral load performed when HCV‐Ag is still positive [13]. Indeed, the present study revealed a non‐negligible number of false‐negative antigen results associated with positive antibodies, which could lead to genuine active infection being ignored (*n* = 34). Adjusting the Ag cut‐off to 0.625 improved PPV, in line with Bui et al. that suggested a 0.7 threshold.

In acute HCV infections, capsid antigen was positive in 71.4% of cases, either alone or in combination with antibody positivity. Thus, detection of capsid antigen associated with clinical (jaundice, vomiting) and biological (increased ALT) criteria would suggest an acute hepatitis C infection. However, false negatives of antigen valence were observed in almost a third of cases. These false negatives were all associated with a low viral load (≤ 5 log_10_ IU/mL) and, in ¾ of cases, with HIV co‐infection. As a reminder, the natural history of chronic HCV infection includes a zenith of VL secondary to infection, followed either by a plateau persistence of VL or by a nadir linked to the establishment of the immune response before a re‐ascension of VL during chronicization of the infection [14, 15]. A sample taken at the time of the ramp‐up phase of the VL or at its nadir may explain the presence of these false negatives.

The main limitation of the Elecsys HCV Duo was reduced sensitivity for low HCV VL (≤ 5 log_10_ IU/mL): only 4.5% in this subgroup, with most false negatives linked to ongoing DAA therapy, HIV‐coinfection, cirrhosis, or dialysis. This aligns with Bertisch et al., who demonstrated that only one‐third of patients with VLs ≤ 3.5 log_10_ IU/mL were capsid antigen‐positive [16]. Consequently, it reinforces that Ag‐negative/Ab‐positive results should not exclude active infection. This highlights the importance of confirmatory HCV RNA testing, particularly in cases with a low VL or where it is suspected.

The absence of fibrosis assessment using the FIB‐4 index represents a limitation of this study, as the required parameters (AST and platelet count) were not consistently available and were not included in the predefined dataset of this non‐interventional study.

Interestingly, the assay identified reinfections in patients with previous HCV cure, with HCVcAg detected in 2 of the 3 observed cases, suggesting that it remains reliable in reinfection cases with high viral loads, but may be limited in cases of low‐level viremia. Although further validation in larger cohorts is required, our results suggest that combined Ag/Ab testing could play a role in identifying reinfection in high‐risk individuals.

The widespread application of the Elecsys HCV Duo test in various clinical departments underscores its versatility and adaptability across healthcare settings. Its use in high‐risk populations, particularly those served by CeGIDD centers and infectious disease departments, aligns with WHO's elimination targets by 2030, especially in settings with limited molecular capacity. Point‐of‐care (POC) HCV RNA testing from capillary whole blood represents a complementary approach to high‐throughput laboratory‐based screening. Their main advantage lies in decentralized, same‐site testing, particularly in populations at high risk of loss to follow‐up, where they facilitate rapid confirmation of active infection and earlier treatment initiation. In contrast, high‐throughput assays such as the Elecsys HCV Duo test are better suited for large‐scale screening in centralized laboratory settings due to their higher processing capacity.

In cases of low‐level viremia, POC RNA testing may provide added diagnostic value compared to antigen‐based assays. However, caution remains warranted near the lower limit of detection, where occasional discordant results may occur. Overall, these approaches should be considered complementary within integrated diagnostic strategies.

Elecsys HCV Duo is a valuable tool for early HCV detection and streamlined patient management, particularly when dual Ag/Ab positivity is present. However, confirmatory RNA remains essential for Ag‐only positives and Ab‐positive/Ag‐negative cases in high‐risk contexts. Future work should focus on improving sensitivity for low VLs, validating adjusted cut‐offs, and assessing cost‐effectiveness in diverse and decentralized screening programmes.

## Author Contributions

C.S. and C.R. conceptualized the study. C.S. supervised the study and administered the project. P.E.G. and C.M.S. curated the data, performed the formal analysis, and drafted the original manuscript. F.Z. supervised patient recruitment, and contributed to critical revision of the manuscript. All authors contributed to reviewing and editing the manuscript.

## Funding

The authors have nothing to report.

## Ethics Statement

The study was approved by the local ethics committee (approval no. 24‐5089). By default, patients provide general consent for the use of their data upon hospital admission and are informed of the study through a transparency portal.

## Conflicts of Interest

The authors declare no conflicts of interest.

## Data Availability

The data underlying this study are not publicly available due to institutional and ethical restrictions, but may be available from the corresponding author upon reasonable request.
